# Adult Intussusception: A Retrospective Review

**DOI:** 10.1007/s00268-014-2759-9

**Published:** 2014-09-06

**Authors:** Hirotaka Honjo, Makio Mike, Hiroshi Kusanagi, Nobuyasu Kano

**Affiliations:** Department of Surgery, Kameda Medical Center, 929 Higashi-cho, Kamogawa, Chiba, 296-8602 Japan

## Abstract

**Background:**

Intussusception is common in children but rare in adults. The goal of this study was to review retrospectively the symptoms, diagnosis, and treatment of intussusception in adults.

**Methods:**

From 1997 to 2013, we experienced 44 patients of intussusception in patients older than 18 years. The patients were divided into enteric, ileocolic, ileocecal, and colocolonic (rectal) types. The diagnosis and treatment of these patients were reviewed.

**Results:**

Of the 44 patients of adult intussusception, 42 were diagnosed with abdominal ultrasonography and abdominal computed tomography. There were 12 patients of enteric intussusception, six patients of ileocolic intussusception, 16 patients of ileocecal type intussusception, and 10 patients of colonic (rectal) intussusception. Among them, 77.3 % were associated with a tumor. Among 12 patients of enteric intussusception, three were associated with a metastatic intestinal tumor, and one was associated with a benign tumor. Among six patients of ileocolic intussusception, two patients were associated with malignant disease. Also, 93.8 % of ileocecal intussusceptions were associated with tumors, 80.0 % of which were malignant. Similarly, 90.0 % of colonic intussusceptions were associated with malignant tumors. Intussusception was reduced before or during surgery in 28 patients. Surgery was performed in 41 patients, and laparoscopy-assisted surgery was performed for ab underlying disease in 12 patients.

**Conclusions:**

Preoperative diagnoses were possible in almost all patients. Reduction greatly benefited any surgery required and the extent of the resection regardless of the underlying disease and surgical site.

## Introduction

Intussusception was first described by Paul Barbette [1] as the proximal portion of the intestine (intussusceptum) invaginating into the distal portion of the intestine (intussuscipiens) in a telescope-like fashion. In 1789, John Hunter described three such patients and coined the term “intussusception” [2]. Sir Jonathan Hutchinson first described reduction of intussusception in 1871 [3].

From 1997 to 2013, more than 17,000 patients were treated surgically in our medical center, but only 44 patients of intussusception were recorded during this period.

Any intestinal condition that changes the normal pattern of peristalsis increases the risk of intussusception [4]. Normal physiologic peristalsis along bowel intussusception promotes extension of the invagination to involve longer segments of the intestine, mesentery, and mesenteric blood vessels [5].

The optimal management strategy for adult intussusception remains controversial and requires consideration of: (1) the frequency of an underlying disease and the need for surgical treatment; (2) the occurrence of malignant disease and excessive surgical treatment; (3) the anatomic location and extent of intussusception; and (4) the presence of any associated inflammation, edema, or bowel ischemia.

In the present study, all patients of intussusception in our department were reviewed in terms of their etiology and treatment. Rectal intussusception is a distinct entity and was not addressed in this study.

## Patients and methods

Between 1997 and 2013, a total of 44 patients of adult (age ≥18 years) intussusception were treated in our institution. Patients were categorized according to the lead point of the intussusception.

- Enteric type: The intussusception is limited to the small intestine.
- Ileocolic type: The ileum passes the ileocolic segment, but the appendix does not invaginate.
- Ileocecal type: The ileocecal portion invaginates into the ascending colon.
- Colocolonic (including colorectal) type: The intussusception is limited to the colon and rectum with no anal protrusion.

We retrospectively assessed the patients regarding the intussusception category, date, patient’s age, sex, and clinical symptoms, and the diagnostic tests that were applied. We also reviewed the surgical procedures, pathologic findings, and postoperative courses.

## Results

### Clinical findings

The youngest patient was 20 years old, and the oldest was 91 years old. (median 70.0 years). The male/female ratio was 20:24.

The most common complaint was abdominal pain [24 patients (54.5 %)]. Vomiting, nausea, diarrhea, and hematochezia were other symptoms (Table 1). In five patients, a tumor was palpable in the abdomen. Twelve patients had symptoms that continued for more than 1 month, and nine patients had acute symptoms (e.g., complete bowel obstruction). Nine patients had had previous surgery.

**Table 1:** Symptoms in adult patients with intussusception

Symptoms and signs	No. of patients	% of patients
Abdominal pain	24	54.5
Vomiting	10	22.7
Diarrhea	8	18.2
Nausea	6	13.6
Melena	6	13.6
Abdominal mass	5	11.4
Abdominal distension	4	9.1
Poor feeding	3	6.8
Epigastralgia	2	4.5
Weight loss	2	4.5
General malaise	1	2.3
Respiratory discomfort	1	2.3
Shock	1	2.3

A total of 43 patients (97.7 %) underwent plain abdominal radiography for evaluation, and 22 patients underwent abdominal ultrasonography (US). Twelve patients had specific findings of intussusception. Abdominal computed tomography (CT) was performed for all patients. Intussusception was diagnosed in 41 patients in a timely fashion. Studies with contrast medium were performed in 13 patients, in 8 of whom intussusception was confirmed. Among those eight patients, three underwent successful reduction. Colonoscopy was performed in 26 patients. In 11 patients, colonoscopy resulted in reduction of the intussusception. These data are summarized in Table 2.

**Table 2:** Patients of preoperative diagnostic studies

Study	Total	Correct diagnosis
Abdominal radiography	43	0
Abdominal CT	44	41
Abdominal US	22	12
Colonoscopy	26	25
Enema	13	8

*CT* computed tomography, *US* ultrasonography

Overall, the diagnosis of intussusception prior to surgery was possible in 42 of 44 patients. Before surgery, 14 intussusceptions were reduced with no complications.

There were 12 (27.3 %) patients who had an enteric intussusception (overlapping patients counted as one patient), 6 (13.6 %) had an ileocolic intussusception, 16 (36.4 %) had an ileocecal intussusception, and 10 (22.7 %) had a colocolonic (colorectal) intussusception.

### Etiology

In all, 34 (77.3 %) patients had organic lesions (tumor-associated). In 5 (11.4 %) patients they were due to postoperative adhesions, and in 5 (11.4 %) they were idiopathic. Pathologic causes were identified in 34 patients (Table 3). Benign lesions were found in nine patients and malignant lesions in 25 patients. The patient group with enteric intussusceptions included five who were postoperative, two whose disease was idiopathic, one with a lipoma, one with an inverted Meckel’s diverticulum, and three with metastatic cancer. Patients with an ileocolic intussusception included two with idiopathic disease, two with a benign tumor (lipoma), and two with malignant lymphoma. Patients with ileocecal intussusception included six patients with cecal cancer and four with a benign tumor (one myxoma of the appendix, one submural lipoma, two cystomyxomas of the appendix). Patients of colocolonic (colorectal) intussusception included nine patients of colon cancer and one with lipoma.

**Table 3:** Causes of adult intussusception

Type	Carcinoma	Other malignant tumor	Benign	Postop.	Idiopathic
Enteric	–	3	2	5	2
Ileocolonic	–	2	2	–	2
Ileocecal	11	–	4	–	1
Colocolonic	9	–	1	–	–

*Postop*. postoperative

### Treatment

Overall, 41 patients underwent surgery. Three patients were treated conservatively because of their poor performance status.

We performed surgery in 11 of 12 enteric intussusception patients, including seven partial resections of the small intestine. One case resolved with observation alone—this patient was not a surgical candidate because of poor performance status. Intraoperative reduction was attempted in 8 of 12 patients with intussusception: Six were successful, but two were unsuccessful because of hard adhesions.

Preoperative reduction was attempted in four of six patients with ileocolic intussusception. It was not successful in any of these patients because the advanced part could not pass through the ileocecal valve. Intraoperative reduction was performed in three of the six patients. We operated on all six patients including five with small bowel resection.

Among 16 patients with ileocecal intussusception, nine experienced reduction preoperatively. In the other seven patients without preoperative reduction, four underwent reduction intraoperatively, one intussusception could not be reduced, and two were reduced with observation alone. We performed surgery for 15 of 16 ileocecal intussusceptions, including 14 patients with a right colectomy. One patient underwent observation alone because of poor performance status.

Among 10 patients with colocolonic (including colorectal) intussusception, five underwent reduction preoperatively. One experienced reduction intraoperatively and two with observation. Two intussusceptions were not successfully reduced. We performed surgery in 9 of 10 patients including eight colectomies. One patient underwent observation only because of poor performance status.

In total, 14 patients underwent intussusception reduction preoperatively and 14 patients intraoperatively (Table 4). Two patients experienced intraoperative perforation, but it did not result in postoperative complications. In 12 patients, laparoscopy-assisted surgery was performed as treatment for the underlying disease. There were no deaths over a 1-month follow-up period.

**Table 4:** Number of successful reductions in adult patients with intussusception

Type	Preoperative	Intraoperative	Spontaneous
Enteric (total 12 patients)	0	6	0
Ileocolic (total 6 patients)	0	3	0
Ileocecal (total 16 patients)	9	4	2
Colocolonic (total 10 patients)	5	1	2

## Discussion

Intussusception is found in 1 % of adult patients with bowel obstruction [6, 7], representing 5–10 % of all patients with intussusception [5, 7]. The average age of affected individuals is 50 years, and the male/female ratio is 1:5.

A literature review of 1214 adult patients with intussusception revealed that 63 % of adult intussusception was tumor-related, 50 % of which were malignant. A malignant tumor was the etiology in 48 % of patients with colocolonic intussusception and in 17 % of those with enteric intussusception [8]. We retrospectively reviewed 16 years of data from patients with intussusception and found that 77.3 % were related to a tumor, 73.5 % of which were malignant. Among them, 11.3 % occurred in a postoperative setting, and 11.3 % were idiopathic. Malignant tumor was the etiology in 90.0 % of patients with colocolonic intussusception and in 25.0 % of those with enteric intussusception.

More than 90 % of adult intussusception patients have distinct causes that are related to the small or large intestine. This is in contrast to the 90 % of childhood intussusception cases that are idiopathic [8]. Etiologies of adult intussusception include tumor- or surgery-related, idiopathic, and “other.” Benign or malignant tumors are the most frequent cause of intussusception in adults.

Many colocolonic intussusceptions were related to primary adenocarcinoma of the colon. Generally, many malignant tumors of the small bowel are metastatic tumors. Postoperative factors are the second most common etiology of intussusception in adults [7, 9].

Most affected adults have pre-diagnosis episodes of intermittent abdominal pain and vomiting [10]. The most common symptoms are due to bowel obstruction and include crampy abdominal pain (71 %), nausea and vomiting (68 %), and sensations of abdominal fullness (45 %) and tenderness (60 %) [8]. The emergence of acute symptoms due to complete intestinal obstruction occurs in fewer than 20 % of patients. Similar results were seen in our study, and the incidence of long-term symptoms was 27.3 %.

It is often difficult to diagnose adult intussusception because the clinical findings are not clear. In the past, colon intussusception was diagnosed with a contrast enema showing a crab claw-like shadow, but the accuracy of preoperative diagnosis was only 20–25 %. Intussusception in adults is often discovered only during exploratory surgery [10].

Abdominal US and CT diagnose intussusception with high sensitivity. CT examination typically reveals a three-layer structure that includes the intestinal wall, its mesenterium, and wrapping intestine. It sometimes reveals the tumor as a lead point [11]. In a recent report by Azar and Berger, abdominal CT accurately diagnosed intussusception in 78 % of patients [7]. CT is the most useful examination for diagnosing intussusception. In our study, US and CT assessments resulted in a combined preoperative diagnosis rate of 95.5 %. CT had an accuracy of almost 100 %, whereas US had an accuracy of only about 50 %. Plain abdominal radiography was of no value.

In adults, it is important to diagnose the organic intussusception lesion to help guide treatment decisions. Enema or colonoscopy examinations can reveal and reduce the intussusception as well as facilitate qualitative diagnosis of the organic lesion [12].

Before the mid-1950s, intraoperative reduction of intussusception followed by removal of the organic lesion was the treatment of choice. Subsequently, it was recommended that colocolonic-type surgical resection be performed without reduction because of the high incidence of malignant disease associated with intussusception [5]. Other investigators then suggested that intussusception in adults should be resected without reduction regardless of the site because malignant disease is highly associated with enteric intussusception [4].

More recently, surgical treatment has been determined according to the length of the affected small intestine in patients with enteric intussusception because of the relatively low incidence of primary malignant disease. In other words, surgery is performed without reducing the intussusception if the affected portion of the small intestine is not extensive. If resection of a long segment of bowel is required, intraoperative reduction is attempted to reduce the length of the resection. Surgical resection without reduction should be limited to primary malignant disease [8].

Sarr et al. [13] at the Mayo Clinic recently questioned the accepted notion that preoperative reduction of intussusception was not recommended because it was associated with malignant disease. Tumor cells are always flowing out of primary lesions, and the reduction of intussusception causes little damage to the intestinal mucosa. Patients with enteric and ileocolic intussusceptions do not have leading points, so there is no need for removal. Preoperative reduction serves several functions, including avoidance of emergency surgery, allowing radical surgery for cancer, and reducing the extent of the intestinal resection. It also allows time for preoperative preparation of the bowel. Careful radiologic or endoscopic evaluation can detect strangulated intussusception that is impossible to reduce preoperatively. These observations have resulted in a shift in the clinical paradigm for reducting the intussusception.

In this study, intussusceptions were reduced before surgery in 14 patients followed by preoperative examination. In another 14 patients, intussusceptions were reduced intraoperatively, thereby decreasing the extent of the resected intestine. We believe that the benefits of intussusception reduction are great unless the local lesion has signs of necrosis. In all, 28 patients (63.6 %) underwent intussusception reduction preoperatively and intraoperatively using this policy. Two patients experienced a small perforation intraoperatively, but it did not result in postoperative complications.

## Conclusions

We reviewed the diagnosis and treatment of 44 adult intussusception patients. Historically, investigators have suggested that it is difficult to diagnose intussusception in adults because it is a subacute disease with nonspecific clinical signs and symptoms. However, current technology allows an easy diagnosis via US, CT, or endoscopy. In addition, the preoperative and intraoperative reduction of intussusception will likely become a standard approach because it offers greater benefits than traditional methods.
